# Beyond Risk: Bacterial Biofilms and Their Regulating Approaches

**DOI:** 10.3389/fmicb.2020.00928

**Published:** 2020-05-21

**Authors:** Musa Hassan Muhammad, Aisha Lawan Idris, Xiao Fan, Yachong Guo, Yiyan Yu, Xu Jin, Junzhi Qiu, Xiong Guan, Tianpei Huang

**Affiliations:** State Key Laboratory of Ecological Pest Control for Fujian and Taiwan Crops & Key Laboratory of Biopesticide and Chemical Biology of Ministry of Education, College of Life Sciences & College of Plant Protection & International College, Fujian Agriculture and Forestry University, Fuzhou, China

**Keywords:** bacterial biofilm, biofilm formation, biofilm risk, biofilm promotion, regulation strategy

## Abstract

Bacterial biofilms are complex surface attached communities of bacteria held together by self-produced polymer matrixs mainly composed of polysaccharides, secreted proteins, and extracellular DNAs. Bacterial biofilm formation is a complex process and can be described in five main phases: (i) reversible attachment phase, where bacteria non-specifically attach to surfaces; (ii) irreversible attachment phase, which involves interaction between bacterial cells and a surface using bacterial adhesins such as fimbriae and lipopolysaccharide (LPS); (iii) production of extracellular polymeric substances (EPS) by the resident bacterial cells; (iv) biofilm maturation phase, in which bacterial cells synthesize and release signaling molecules to sense the presence of each other, conducing to the formation of microcolony and maturation of biofilms; and (v) dispersal/detachment phase, where the bacterial cells depart biofilms and comeback to independent planktonic lifestyle. Biofilm formation is detrimental in healthcare, drinking water distribution systems, food, and marine industries, etc. As a result, current studies have been focused toward control and prevention of biofilms. In an effort to get rid of harmful biofilms, various techniques and approaches have been employed that interfere with bacterial attachment, bacterial communication systems (quorum sensing, QS), and biofilm matrixs. Biofilms, however, also offer beneficial roles in a variety of fields including applications in plant protection, bioremediation, wastewater treatment, and corrosion inhibition amongst others. Development of beneficial biofilms can be promoted through manipulation of adhesion surfaces, QS and environmental conditions. This review describes the events involved in bacterial biofilm formation, lists the negative and positive aspects associated with bacterial biofilms, elaborates the main strategies currently used to regulate establishment of harmful bacterial biofilms as well as certain strategies employed to encourage formation of beneficial bacterial biofilms, and highlights the future perspectives of bacterial biofilms.

## Introduction

It is now understood that about 40–80% of bacterial cells on earth can form biofilms (Flemming and Wuertz, 2019). The formation of biofilms was detrimental in several situations (Donlan and Costerton, 2002; Dobretsov et al., 2006; Coughlan et al., 2016). For example, in food industries, pathogenic bacteria are able to form biofilms inside of processing facilities, leading to food spoilage, and endangering consumer’s health (Galie et al., 2018). In hospital settings, biofilms have also been shown to persist on medical device surfaces and on patient’s tissues causing persistent infections (Dongari-Bagtzoglou, 2008; Percival et al., 2015). In view of the serious impact of biofilms on human health and other aspects, researchers and the public have long focused on prevention and control of the harmful biofilms.

Despite the negative impacts, bacterial biofilms may also have beneficial effects (Rosche et al., 2009). That is, the formation of bacterial biofilms is often important in agricultural and other industrial settings (Bogino et al., 2013; Berlanga and Guerrero, 2016). These beneficial biofilms are currently used as biological control agents against phytopathogens and biofertilizers to enhance crop production (Timmusk et al., 2017), for bioremediation treatment of hazardous pollutants (Irankhah et al., 2019), for wastewater treatment (Ali et al., 2018), for protection of marine ecosystem (Naidoo and Olaniran, 2013), and for prevention of corrosion (Jayaraman et al., 1997; Martinez et al., 2015). Although biofilms can be beneficial to agriculture and industry, people’s understanding of the harmfulside of biofilms has been far better than the benefits for decades. Therefore, the beneficial aspects of biofilms will have great development prospects in the future.

Biofilms are complex surface attached communities of microorganisms held together by self-produced polymer matrixs mainly composed of polysaccharides, secreted proteins, and extracellular DNAs (Tremblay et al., 2013). A biofilm can consist of a single microbial species or a combination of different species of bacteria, protozoa, archaea, algae, filamentous fungi, and yeast that strongly attach to each other and to biotic or abiotic surfaces (Tomaras et al., 2003; Bogino et al., 2013; Silva et al., 2014; Costa-Orlandi et al., 2017; Raghupathi et al., 2017). The ability of microorganisms to develop biofilms has been shown to be an adaptable attribute of microbes (Koczan et al., 2011). The formation of biofilm appears to be an age-old survival mechanism that provides microorganisms with better options compared to their planktonic cells (Dang and Lovell, 2016), including stronger ability to grow in oligotrophic environments (Bowden and Li, 1997), greater access to nutritional resources (Dang and Lovell, 2016), improved survival to biocides (Flemming et al., 2016), enhanced organism productivity and interactions (Roder et al., 2018), as well as greater environmental stability (Dang and Lovell, 2016). It can be seen that biofilms provide protection for bacteria and make them more suitable for the external environment under certain conditions.

Generally, bacterial biofilm formation relies on the interaction between the bacterial cells, the substrates and the surrounding media (Van Houdt and Michiels, 2010). And the formation of bacterial biofilms is a multi-step process starting with reversible attachment to surfaces aided by intermolecular forces and hydrophobicity, and then progress to extracellular polymeric substances (EPS) production which enable the cells to permanently adhere to a surface (Dunne, 2002; Bogino et al., 2013; Caruso et al., 2018). More specially, there are five main phases involved in the biofilm formation process: reversible attachment, irreversible attachment, EPS production, maturation of biofilm, and dispersal/detachment (Stoodley et al., 2002; Toyofuku et al., 2016). However, the expression and regulation mechanisms of different species of bacteria on various phases of biofilms are quite diverse. Before fully understanding the formation process of all bacterial biofilms, researchers still have a long way to walk.

Nowadays, a variety of approaches, which were mostly concerned with interference against bacterial attachment, signal transduction (quorum sensing interference), and disruption of biofilm architecture, have been applied to inhibit formation of harmful biofilms (Chung and Toh, 2014; Galie et al., 2018). In addition, formation of beneficial biofilms can be encouraged through manipulation of adhesion surfaces, quorum sensing (QS) signals and environmental conditions (Upadhyayula and Gadhamshetty, 2010; Renner and Weibel, 2011; Mangwani et al., 2016). Compared with the researches that promote the formation of beneficial biofilms, the investigation on the prevention and control of harmful biofilms is much deeper.

In order to have a comprehensive understanding of bacterial biofilms beyond risk, this review describes the events involved in bacterial biofilm formation, lists the negative and positive aspects associated with bacterial biofilms, elaborates the main strategies currently used to regulate establishment of harmful bacterial biofilms as well as certain strategies employed to encourage formation of beneficial bacterial biofilms, and highlights the future perspectives of bacterial biofilms.

## Risks of Bacterial Biofilms

Bacteria are able to colonize and form biofilms on virtually all kinds of surfaces, including natural and synthetic surfaces (Hall-Stoodley et al., 2004; Sweet et al., 2011). Biofilms are responsible for chronic illness and nosocomial infections, industrial pipe fouling, spoilage of foods, contamination of sea food, and dairy products as well as ship hull fouling (Zottola and Sasahara, 1994; Schultz et al., 2011; Abdallah et al., 2014; Khatoon et al., 2018). Therefore, the harmful effects of biofilms on human society are manifold.

### Healthcare Issues

In the healthcare settings, biofilms have been shown to develop on medical device surfaces, dead tissues (e.g., sequestra of bones), and inside living tissues (e.g., lung tissue, teeth surfaces; Alav et al., 2018). They may develop on the surface of biomedical devices such as catheters, prosthetic heart valves, pacemakers, breast implants, contact lenses, and cerebrospinal fluid shunts (Table 1; Hall-Stoodley et al., 2004; Wu et al., 2015). Both Gram-positive and Gram-negative bacteria may attach to and develop biofilms on the surfaces of these devices, but the most frequently reported biofilm forming bacteria are *Staphylococcus aureus, Staphylococcus epidermidis* and *Pseudomonas aeruginosa* (Hall-Stoodley et al., 2004; Shokouhfard et al., 2015; Khatoon et al., 2018; Pakharukova et al., 2018). It is estimated that about two-thirds of indwelling devices related infections are caused by the staphylococcal species (Khatoon et al., 2018). Bacterial biofilms can also develop in health care water distribution systems. *P*. *aeruginosa* can form biofilms on inner surfaces of metal pipes in hospital water system (Loveday et al., 2014). In addition, biofilm forming bacteria contribute to a lot of life-threatening infections and diseases in humans such as cystic fibrosis (CF), otitis media, periodontitis, infective endocarditis (IE), chronic wounds, and osteomyelitis (Southey-Pillig et al., 2005; Akyildiz et al., 2013; Masters et al., 2019). More specially, *P. aeruginosa* biofilm can cause severe pulmonary infections in patients with CF (Southey-Pillig et al., 2005; Rabin et al., 2015); *Haemophilus influenza* biofilm is among the causative agents of otitis media (Akyildiz et al., 2013; Bjarnsholt, 2013); Periodontitis, an infection of the gums that damages the soft tissues as well as bones supporting the teeth, is normally caused by the biofilms of *Pseudomonas aerobicus* and *Fusobacterium nucleatum* (Jamal et al., 2018); The hypothesis that IE, notoriously difficult to treat, is a biofilm infection explains its resistance to antimicrobials and why surgical disruption and removal of the biofilm improves the chance of cure (Elgharably et al., 2016); *P. aeruginosa* biofilm is also usually formed on chronic wound (Rabin et al., 2015); and Chronic osteomyelitis is a biofilm infection, where microorganisms adhere to dead bone (Zimmerli and Sendi, 2017). It is believed that biofilm-related organisms account for more than 65% of all microbial infections and exhibit high resistance to antimicrobial agents and components of the host defense system (both innate and adaptive; Jamal et al., 2018; Ciofu and Tolker-Nielsen, 2019). Herein, biofilms have huge impacts on human healthcare.

**TABLE 1:** Biofilm forming bacteria on medical devices.

**Medical devices**	**Biofilm-forming bacteria**	**References**
Contact lenses	*P. aeruginosa*, *S. aureus, S. epidermidis, S. saprophyticus, Klebsiella* spp.	El-Ganiny et al., 2017
Central venous catheters	Coagulase-negative *Staphylococci*, *S. aureus, Enteric* Gram-negative *Bacilli*	Gominet et al., 2017
Urinary catheters	*S. aureus, Enterococcus faecalis, P. aeruginosa*	Murugan et al., 2016
Peritoneal dialysis catheters	*S. epidermidis, P. acnes, S. warneri, S. lugdunensis, R. mucilaginosa*	Pihl et al., 2013
Mechanical heart valves	*Streptococcus* spp., *S*. *aureus*, *S. epidermidis*, Gram-negative *Bacillus, Enterococcus*	Jamal et al., 2018
Cerebrospinal fluid shunts	*S*. *aureus*, *S. epidermidis, Enterococcus faecalis, Enterococcus faecium*	Bayston et al., 2012
Breast implants	*S. epidermidis*, Coagulase-negative *Staphylococci, Propionibacterium acnes*	Pajkos et al., 2003; Rieger et al., 2013
Orthopaedic implants	*S. aureus, S. epidermidis, P. aeruginosa, E. coli, S. haemolyticus*	Arciola et al., 2015
Dental implants	Gram-positive cocci, Actinomyces spp., Gram-negative anaerobic oral bacteria	Dhir, 2013; Veerachamy et al., 2014
Voice prostheses	*S. aureus, P. aeruginosa, Klebsiella* spp., *Enterobacter*spp*., R. dentocariosa*, and *Proteus* spp.	Somogyi-Ganss et al., 2017
Cardiac pacemakers	*S. aureus, S. epidermidis*	Santos et al., 2011
Intrauterine devices	*E. coli, Streptococcus agalactie, S. aureus, Enterococcus faecalis, Lactobacillus* spp., *Prevotella* spp., *Porphyromonas* spp., *Bacteroides, Fusobacterium* spp.	Pal et al., 2005
Biliary stents	*Pseudomonas, Citrobacter, Klebsiella, Staphylococcus, Enterococcus, Aeromonas, Proteus, Enterobacter*	Vaishnavi et al., 2018

### Plant Diseases

Biofilm-related diseases have also been reported in agricultural settings. *Xanthomonas citri* biofilm can cause plant diseases like pierce’s disease of grapevines and citrus canker (Ference et al., 2018; Kyrkou et al., 2018). Strong biofilm producer *Xylella fastidiosa* can also cause pierce’s disease of grapevines by blocking the plant vasculature (Rudrappa et al., 2008; Kyrkou et al., 2018). Biofilms have also been implicated in brown spot disease of bean leaves caused by *P*. *syringae* pv. *syringae* (Monier and Lindow, 2004; Danhorn and Fuqua, 2007). Similarly, *P. aeruginosa* biofilm on the roots of *Arabidopsis* and *Ocimum basilicum* (sweet basil) can cause mortality in a short time. *Ralstonia solanacearum*, an important plant pathogenic bacterium reported to form biofilms on the surfaces of xylem vessels, cause bacterial wilt disease in plants (Yao and Allen, 2007; Mori et al., 2016). It is becoming obvious that bacteria can form biofilms when colonizing different plant surfaces.

### Food Safety and the Food Industry

Within the food industry, biofilms can occur on surfaces contacting with, or without foods (Zottola and Sasahara, 1994; Kumar and Anand, 1998). Biofilms are responsible for about 60% of foodborne outbreaks (Han et al., 2017). Therefore, the presence of biofilms in food processing environments poses significant risk to food safety and the food industry (Galie et al., 2018). In the food processing environments, contaminants mostly come from the surrounding air, equipments, or food surfaces (Kumar and Anand, 1998). Then biofilms growing in food processing environments may lead to spoilage of food, which in turn can cause serious public health risk to consumers and serious economic consequences (Coughlan et al., 2016; Galie et al., 2018). The most common biofilm forming foodborne pathogens and spoilage organisms are *Listeria monocytogenes* (a ubiquitous species that can cause abortion in pregnant women and other complications in immunocompromised individuals; Galie et al., 2018), *Salmonella* spp. (a major cause of foodborne diseases which can lead to Reiter’s syndrome or even death; Ajene et al., 2013; Wirtanen and Salo, 2016), *Escherichia coli* 0157:H7 (a strain which is responsible for hemorrhagic colitis; Wirtanen and Salo, 2016), *Pseudomonas* spp. (a ubiquitous spoilage organism which produces proteases with negative impacts on foods; Rajmohan et al., 2002), *Vibrio parahaemolyticus* (its infection most commonly associated with consumption of undercooked seafood; Yeung and Boor, 2004), *Clostridium perfringens* (a species producing different toxins; Wirtanen and Salo, 2016), *Campylobacter jejuni* (a major cause of human bacterial gastroenteritis; Wirtanen and Salo, 2016), *Bacillus* spp. (a species secreting toxins that can cause diarrhea and emetic syndrome; Galie et al., 2018), *S. aureus* (a species secreting enteric toxins that cause foodborne intoxications; Argudin et al., 2010), *Shewanella putrefaciens* (a species producing volatile sulfides, amines, and trimethylamine; Bagge et al., 2001), *Cronobacter* spp. (a genus mostly causing infections in infants and immunocompromised individuals; Wirtanen and Salo, 2016), and *Geobacillus stearothermophilus* (a common contaminant of dairy products; Table 2; Burgess et al., 2017). These organisms even can establish multi-species biofilms, which are more stable and difficult to control (Bagge et al., 2001; Coughlan et al., 2016; Han et al., 2016; Wirtanen and Salo, 2016; Galie et al., 2018). Biofilms are also responsible for serious technical challenges of food industry in that they may prevent the flow of heat across equipment surfaces, increase the fluid frictional resistance at the surfaces, and promote the corrosion rate of the surfaces, leading to loss of production efficiency (Chmielewski and Frank, 2003; Meesilp and Mesil, 2019). In a word, biofilms have the danger of direct contamination with pathogenic bacteria in the food industries, as well as the risk of contamination of instruments and equipment.

**TABLE 2:** Representative of foodborne bacteria that can form biofilms.

**Foodborne bacteria**	**Growing substrate**	**Spoiledfood**	**References**
*Listeria monocytogenes*	Wastewater pipes, floors, conveyor belts, rubber seals, elastomers, and stainless steel	Dairy products, melons, coleslaw, ready to eat meat products and ready to eat fish products	Wirtanen and Salo, 2016
*Pseudomonas* spp.	Conveyor belts, floors, drains, slicing, and milking machine	Dairy products, red meat, and poultry	Korber et al., 2009; Møretrø and Langsrud, 2017
*Bacillus cereus*	Stainless steel, plastic, soil, and glass wool	Sprouted seeds, fruit juices, fried rice, pasta dishes, meat products, vegetables, and milk products	Korber et al., 2009; Wirtanen and Salo, 2016
*Salmonella*	Stainless steel, elastomers, concrete, glass, and food surfaces (like lettuce and tomato)	Poultry, pig, cow meats, and dairy products	Wirtanen and Salo, 2016
*Escherichia coli*	Stainless steel surfaces, food contact surfaces	Dairy products, fermented meat sausage, meat, poultry, fish products, drinks, and vegetables	Wirtanen and Salo, 2016
*Clostridium*	Multi-species biofilm	Dairy products, fish, cattle meat, poultry, vegetables, honey, and canned food	Wirtanen and Salo, 2016
*Cronobacter* spp.	Powder service and powder packaging rooms, spray-drying areas, and evaporator rooms	Dairy products, vegetables, grains, bread, herbs, sausages, spices, and meat	Wirtanen and Salo, 2016
*Staphylococcus*	Stainless steel, plastics (such as polystyrene and polypropylene), and glass	Dairy products, ready to eat meat products, ready to eat fish and seafood products, and ready to eat dairy products	Wirtanen and Salo, 2016

### Drinking Water Distribution Systems

Biofilms are the predominant mode of microbial growth within the drinking water distribution systems (Mahapatra et al., 2015; Liu et al., 2016). It is well documented that biofilms represent one of the major problems in drinking water distribution systems (Douterelo et al., 2016; Prest et al., 2016). The consumption of contaminated water with pathogenic biofilms has been linked to human infections and waterborne outbreak (Angles et al., 2007; Prest et al., 2016). And the major biofilm producing bacteria in drinking water are *P. aeruginosa*, *Campylobacter jejuni*, *Legionella pneumophila, Mycobacteria, Aeromonas hydrophila*, and *Klebsiella pneuminiae* (Prest et al., 2016; Chan et al., 2019). Since bacterial cells can attach and develop biofilms on the inner surfaces of piping systems from which cells could be detached into the bulk water, they may cause biocorrosion of pipes, undesirable water quality changes affecting color, taste, turbidity and odors, and reduction of heat exchange efficiency (Prest et al., 2016). More specially, the major biofilm producing bacteria known to promote corrosion of metals are sulfate-reducing bacteria, sulfur-oxidizing bacteria, iron-oxidizers, iron-reducers, and manganese-oxidizers (Kip and van Veen, 2015). All in all, biofilms can affect the safety of drinking water and adversely affect water pipelines.

### Marine Biofouling

Marine biofouling portrays the undesirable accumulation of organisms on any natural or man-made objects exposed to seawater (Dobretsov et al., 2013). Common examples of marine substrates include ship hulls and oil or gas installations. Biofouling has been a major challenge in the naval industry and for civilian oceangoing ships (Hopkins and Forrest, 2010; Schultz et al., 2011). Bacteria are among the early microorganisms to settle and colonize substrates in the marine environment and may subsequently facilitate attachment and colonization of larger fouling organisms, such as algae, mussels, and barnacles. Herein, marine biofilms cause biofouling (de Carvalho, 2018). Generally, accumulation of biofoulers by biofilms on ship hulls can increase the hydrodynamic drag of the ships, which causes challenges for shipping industry, including speed reduction, an increase in cleaning time, and greater fuel consumption (Schultz et al., 2011; Demirel et al., 2017). In addition, biofouling of ship hulls has been considered as an important vector for the spread of invasive marine species to new habitats. These transported organisms can adversely affect native species through competition and predation (Minchin and Gollasch, 2003). Therefore, biofilms will affect the cost of ship usage and the balance of marine environment.

## Benefits of Bacterial Biofilms

Despite their negative impacts in ecosystems, biofilms have positive effects in agricultural, and other industrial settings (Bogino et al., 2013; Berlanga and Guerrero, 2016). That is, they could be used for plant protection, bioremediation, wastewater treatment, prevention of corrosion, and other useful applications (Table 3; Morikawa, 2006; Singh et al., 2006; Edwards and Kjellerup, 2013; Naidoo and Olaniran, 2013; Singh et al., 2019). As researches progress, the beneficial aspects of biofilms will receive more attention.

**TABLE 3:** Examples of beneficial applications of bacterial biofilms.

**Applications**	**Purposes**	**References**
Biofertilizer/biocontrol	Plant growth promotion and protection against phytopathogens	Das et al., 2017
Bioremediation	Transformation of hazardous pollutants to harmless substances	van Dillewijn et al., 2009
Wastewater treatment	Removal of contaminants from wastewater	Yamashita and Yamamoto-Ikemoto, 2014
Microbial fuel cells (MFCs)	Electricity generation, biohydrogen production, and wastewater treatment	Ali et al., 2018
Anticorrosion	Corrosion inhibition for metals	Zuo, 2007
Bioleaching	Extraction of metals from their ores e.g., copper, nickel, cobalt, zinc	Siezen and Wilson, 2009
Biofilm reactor	Production of fermented products and wastewater treatment	Morikawa, 2006
Human gut microbiome	Production of vitamins, degradation of toxic compounds and conversion of complex sugar polymers into short-chain fatty acids	de Vos, 2015

### Plant Protection Agents

Biofilm formation triggers a number of beneficial effects such as biocontrol and symbiosis. In plants, bacterial biofilms can be formed on the surfaces of leaves, roots, and stems (Morikawa, 2006; Bogino et al., 2013; Schirawski and Perlin, 2018). Biofilm-forming rhizobacteria can act as biocontrol agents due to their successful colonization of plants surfaces (Bais et al., 2004; Vejan et al., 2016). Such rhizobacteria belong to *Bacillus, Pseudomonas, Streptomyces, Serratia*, and *Stenotrophomonas* (Arrebola et al., 2019). Beneficial bacteria could also be used as biofertilizers to promote plant growth through nitrogen fixation, mineral nutrient uptake, phytohormone production, and disease suppression as well as protection from both biotic and abiotic stresses (Bais et al., 2004; Khan et al., 2018).

The genus *Bacillus* consists of important plant-associated strains employed for both biocontrol and plant growth promotion (Morikawa, 2006). For example, *Bacillus subtilis* is a prominent rhizobacterium which is used as an efficient biocontrol and growth promotion agent to protect plants from bacterial and fungal pathogens due to the formation of robust biofilms and the production of several antagonistic metabolites (Bais et al., 2004; Morikawa, 2006). These metabolites mainly include lipopeptides (such as surfactin, iturin, and fengycins), bacteriocins and siderophores (Meena and Kanwar, 2015; Fira et al., 2018). The colonization by *B. subtilis* in plant roots is associated with surfactin production and biofilm formation, and the surfactin confers protection of plants from pathogen *Pseudomonas syringae* infection (Bais et al., 2004; Stein, 2005).

A large number of root-associated *Pseudomonas* spp. can act as biocontrol agents. They can produce a wide range of antagonistic compounds, including cyclic lypopeptides, pyrrolnitrin, and phenazines, to prevent proliferation of plant pathogens (Arrebola et al., 2019). For example, priming the seeds with phenazine-producing *Pseudomonas chlororaphis* can provide protection of barley and oats against seed borne diseases (Chin-A-Woeng et al., 2003); *Pseudomonas putida* 06909 attaches and colonizes the hyphae of citrus root rotting fungus *Phytophthora parasitica* by feeding on its exudates and then develop a biofilm around the citrus roots, which prevents further proliferation of the fungus (Steddom et al., 2002; Pandin et al., 2017); and Peanut rhizosphere biofilm formation by *Paenibacillus polymyxa* provides protection of peanut plants against crown root rot disease caused by *Aspergillus niger* (Haggag and Timmusk, 2008). Rhizhosphere colonization of beneficial biofilms usually offer excellent plant growth promotion and protection against phytopathogens (Das et al., 2017).

### Bioremediation

Bioremediation is a process that employs living organisms or their derivatives for treatment of hazardous substances from the environment (soil, water, and air) into lesser or harmless compounds (van Dillewijn et al., 2009). It is thought to be a better option than conventional physical and chemical remediation measures with regard to cost and environmental safety (Singh et al., 2006). Moreover, biofilm-mediated remediation methods exhibit higher efficiency in transforming toxic wastes because of improved bioavailability of the pollutants to degrading organisms and enhanced adaptability of degrading microorganisms to different toxic compounds (Upadhyayula and Gadhamshetty, 2010). The process usually occurs as part of microbial metabolism and relies on the enzymatic attack by microbes to convert environmental pollutants into innocuous products (Karigar and Rao, 2011). Numerous microorganisms are capable of transforming wide varieties of environmental pollutants into non-toxic forms (van Dillewijn et al., 2009). Microbial bioremediation can be at the site of contamination (*in situ*) or off the place of contamination (*ex situ*; Kapley and Purohit, 2009). It can be achieved through the incorporation of limiting nutrients and electrons (biostimulation) or by the addition of microbes at the polluted sites (bioaugmentation) to promote the transformation process (Mangwani et al., 2016).

Compared with their planktonic counterparts, microorganisms living in biofilms display greater tolerance to contaminants, higher chance of survival and adaptation as well as stronger abilities to decompose different pollutants through catabolic pathways (van Dillewijn et al., 2009). Biofilm forming bacteria can efficiently be used in the remediation process as cells are encased within a matrix of EPS, which offers protection against several environmental hazards (Mangwani et al., 2016). In addition, biofilms provide an essential habitat which encourages intercellular gene transfer, cellular communication with QS, cohesion and metabolite diffusion as well as bacterial chemotaxis characteristic (Santos et al., 2018).

Biofilm mediated remediation can harbor diverse species of both aerobic and anaerobic bacteria that often use the degradation of pollutants as an energy source (Rodriguez-Martinez et al., 2006). During aerobic degradation, bacteria can use oxygen as final electron acceptor to breakdown toxic contaminants into innocuous products, mainly carbon dioxide, and water (Edwards and Kjellerup, 2013; Azubuike et al., 2016). In anaerobic conditions, electron acceptors such as nitrate and sulfate can play the role of oxygen to transform contaminants into less toxic or harmless substances and the byproduct may depend on the electron acceptor (Rodriguez-Martinez et al., 2006).

Currently, there is an increasing interest in the use of bacterial biofilms mediated remediation for removal of different kinds of environmental pollutants like oil spills, persistent organic pollutants (such as polycyclic aromatic hydrocarbons, polychlorinated biphenyls, and polychlorinated ethenes), heavy metals, dyes, explosives, pesticides, and pharmaceutical products (Edwards and Kjellerup, 2013). Hence, biofilm-mediated bioremediation is employed in the industry for remediation of contaminated soil and groundwater (Edwards and Kjellerup, 2013). *Pseudomonas, Dehalococcides, Arthrobacter, Bacillus, Alcanivorax, Cycloclasticus, Burkholderia*, and *Rhodococcus* can remediate these pollutants (Dasgupta et al., 2013; Yoshikawa et al., 2017). It is likely that more and more bacterial biofilms will be applied to bioremediation.

### Wastewater Treatment

Nowadays, water contamination caused by industrialization, population growth, and urbanization has become a major global threat (Daud et al., 2017). Wastewater is composed of a broad range of organic and inorganic contaminants originating from storm water, agriculture, industry, domestic, and commercial sewage (Naidoo and Olaniran, 2013). The treatment of wastewater is essential to the protection of aquatic ecosystems and public health (Naidoo and Olaniran, 2013). There are several physicochemical processes for wastewater treatment such as coagulation/flocculation, membrane filtration systems, and electrochemical treatment (Kobya et al., 2009; Francis et al., 2016; Favero et al., 2018). Even though these processes provide effectiveness, they experienced difficulty in removing organic matters in that the main components in the conventional water treatment systems are disinfection and filtration (Hlihor et al., 2017). Bacterial communities have been employed to neutralize and degrade organic and inorganic compounds in wastewater through the use of biofilm-based wastewater treatment technology. Removal of excess nutrients from wastewater is also imperative to avoid aquatic eutrophication which leads to anoxia (Yamashita and Yamamoto-Ikemoto, 2014). The basic nutrients present in wastewater are mostly nitrogen and phosphorous (Yamashita and Yamamoto-Ikemoto, 2014). Hence, among the bacterial species used in wastewater treatment are often denitrifying species or those capable of neutralizing phosphorous (Zielinska et al., 2016).

Biologically active carbon (BAC) process, one of the water treatment biotechnologies, uses granular activated carbon (GAC) as a water filtration media to physically remove water-borne disease causing microorganisms, organic matter and in organic substances (Shirey et al., 2012). After the GAC media particles became exhausted, the rough porous surfaces of this GAC are amenable to colonization of bacteria and formation of bacterial biofilms, which degrade phosphorous and nitrogen-containing compounds, organic carbon as well as other entrapped contaminants in the influent water (Simpson, 2008). Currently, biofilm reactors are developed for wastewater treatment such as membrane reactors, moving beds, fluidized beds, and rotating contactors (Huang et al., 2019).

Biofilms can also be used in bioelectrochemical systems (BESs; Upadhyayula and Gadhamshetty, 2010). BESs are bioreactors that utilize microorganisms as catalysts to convert the energy present in organic wastes into electrical energy (Bajracharya et al., 2016). BESs can facilitate wastewater treatment, bioremediation as well as production of power, fuels and chemicals (Ren et al., 2019). BES electrode surface remodeling has been considered an effective technique to improve the performance of BESs (Ren et al., 2019). Microbial fuel cells (MFCs) are a type of BESs that offer another approach for wastewater treatment in an inexpensive way (Ren et al., 2008; Mei et al., 2017). All sorts of wastewater containing compounds degradable by bacteria can be treated by MFCs, including brewery effluent, petroleum contaminants, domestic wastes, food processing waste, swine manure slurry, landfill leachate, and so on (Franks and Nevin, 2010; Gude, 2016a). MFC uses bacteria in the waste as a biocatalyst to convert the chemical energy present in the wastes to electrical energy using oxidation-reduction reactions (Franks et al., 2010; Angelaalincy et al., 2018). MFCs are primarily made of an anode and a cathode separated by a semi-permeable membrane (Franks et al., 2010). The use of MFCs for wastewater treatment needs a design which permits the passage of wastewater through the cell over the anode surface. Bacterial attachment, colonization and biofilm development occur on the anode surface, the bacteria then oxidize the substrate in wastewater to produce electrons and protons. The electrons released during oxidation flow to the cathode via an electrical circuit to generate current (Ali et al., 2018; Singh et al., 2019). At the cathode, electron acceptors (usually oxygen) react with protons, and electrons to generate water vapor-like reduced compounds (Cao et al., 2019). Most of the MFCs configurations can achieve chemical oxygen demand (COD) removal efficiencies at wastewater treatment (Liu et al., 2004; Min et al., 2005; Gude, 2016b). Liu et al. (2004) were the first to initiate the application of MFCs, which reached 80% of COD removal efficiency from real domestic wastewater with a maximum electrical power generation of 26 mW/m^2^ using a single-chamber MFC. Min et al. (2005) had demonstrated that COD and ammoniacal nitrogen (NH^+^_4_-N) removal are 86% and 83% with a maximum power output of 45 mW/m^2^ when a swine wastewater is treated with a dual-chambered MFC.

### Prevention and Control of Corrosion

Corrosion has now been widely acknowledged as a big problem in drinking water distribution systems, medical, marine, and food processing industry (Prest et al., 2016; Jia et al., 2017; Guo et al., 2018). Both chemical and biological factors can accelerate the rate of corrosion (Kip and van Veen, 2015). Obviously, the activities of microbes on surfaces of metallic materials can either inhibit or promote corrosion (Zuo, 2007). Different strategies, including protective coatings, biocides, cathodic protection and corrosion inhibitors, have been developed to prevent corrosion (Zuo, 2007). However, more recently there has been increased interest in the use of beneficial bacterial biofilms to prevent corrosion because of their effectiveness, cost effective and nature friendly behavior (Zuo, 2007; Guo et al., 2018). The potential strategies may involve: (i) removal of corrosive substances such as oxygen by aerobic bacteria through respiration; (ii) inactivation of corrosive inducing bacteria like sulfate reducing bacteria by inhibitory antimicrobial compounds secreted within biofilms; (iii) production of protective coats such as γ-polyglutamate by biofilms; and (iv) biofilms formation serving as a diffusion barrier to hinder dissolution of metals (Zuo, 2007; Guo et al., 2018). A gramicidin-S-producing *Bacillus brevis* biofilm has been reported to curtail the rate of corrosion in mild steel by suppressing the growth of sulfate-reducing bacterium *Desulfosporosinus orientis* and the iron-oxidizing bacterium *Leptothrix discophora* SP-6 (Zuo et al., 2004). Also, the antimicrobial compounds indolicidin, bactenecin and probactenecin produced by genetically engineered *B. subtilis* biofilm can suppress metal corrosion by inhibiting the growth of *D. vulgaris* and *D. gigas* (sulfate-reducing bacteria; Zuo, 2007). Although, both aerobic and anaerobic biofilms are able to reduce corrosion rates on the surfaces of different materials, the aerobic biofilms remarkably suppress metal corrosion, which suggests that oxygen consumption can further enhance corrosion protection (Kip and van Veen, 2015). Anticorrosive approach via beneficial biofilms has been successfully reported for stainless steel, carbon steel, copper, and aluminum (Guo et al., 2018). The use of bacterial biofilms for prevention and control of corrosion is a relatively new direction and deserves special attention.

## Biofilm Formation Process

**FIGURE 1:**
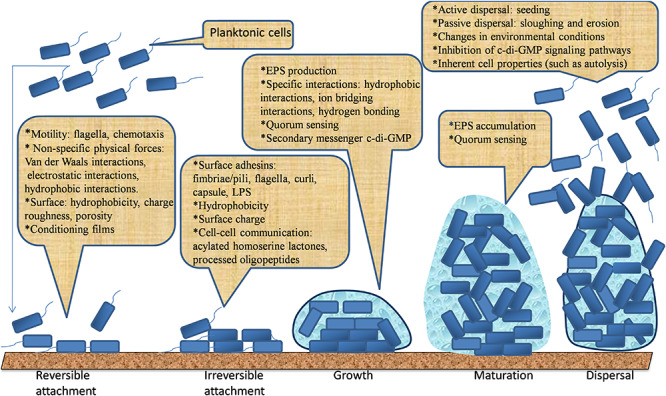
The five main phases leading to the development and dispersal of biofilm.

Bacteria form biofilms in response to environmental stresses such as UV radiation, desiccation, limited nutrients, extreme pH, extreme temperature, high salt concentrations, high pressure, and antimicrobial agents. Herein, the events leading to bacterial biofilm formation are complex (O’Toole et al., 2000; Hall-Stoodley et al., 2004; Lopez et al., 2010; Galie et al., 2018). It is generally believed that biofilm formation starts with a reversible attachment of bacteria onto a surface, followed by the irreversible attachment, usually aided by adhesive structures of bacteria and short-range interactions. Their reversible attachment is progressed through the production of EPS. Later, they develop into an organized structure entrapped in an EPS matrix. Finally, bacterial cells can escape from the mature biofilm and disperse into the environment to colonize new niches (Berne et al., 2015; Hoffman et al., 2015; Limoli et al., 2015; Toyofuku et al., 2016). These phases of biofilm formation are illustrated in Figure 1. Five main phases leading to the development of free-living planktonic life form into a sedentary “biofilm” lifestyle are discussed below.

### Reversible Attachment

Bacterial attachment is the initial step of biofilm formation. It begins with the favorable interaction between a few planktonic cells and substrate surfaces. The bacteria must be transported to the surfaces by Brownian motion, sedimentation, or convection (Palmer et al., 2007). Chemotaxis is the directed movement of bacterial cells toward a nutrient source or chemoattractants (e.g., amino acids and sugars) along a concentration gradient in mobile fluids. It occurs in virtually all microorganisms and can facilitate bacterial growth on surfaces by enabling cell-surface interactions (Vladimirov and Sourjik, 2009; Porter et al., 2011). Once the cells reach a surface, the interaction between the cell surfaces and the conditioned surface depends on the net sum of repulsive or attractive forces generated between the two surfaces. If the attractive forces are greater than the repulsive forces, the bacteria will attach to the surface and vice versa (Dunne, 2002; Carniello et al., 2018). This initial attachment is achieved through the effects of non-specific physical forces such as electrostatic forces, hydrophobic interactions and Lifshitz–van der Waals interactions (Dunne, 2002; Carniello et al., 2018). Bacterial attachment has been interpreted within the scope of the classical Derjaguin, Verwey, Landau, and Overbeek (DVLO) DVLO theory, the extended DVLO model, and the thermodynamic approaches (Perni et al., 2014; Zhang et al., 2015; Carniello et al., 2018). These theories describe attachment as the result of a balance between attractive Lifshitz–van der Waals interactions and repulsive forces, based upon electrostatic forces (Morra and Cassinelli, 1997; Rijnaarts et al., 1999), in addition to hydration forces (Jucker et al., 1998; Hermansson, 1999). In general, the reversible bacterial attachment to a surface involves deposition of a bacteria to a substrate in such a way that the bacteria remain in a two dimensional Brownian motion and can be easily detached from the surface by either bacterial mobility or shearing effects of a fluid flowing over the surface (Li and Tang, 2009; Carniello et al., 2018).

Both inert and biological surfaces can be used for initial bacterial attachment. In fact, any substance coming into contact with bacterial suspension is considered to be a substrate for biofilm growth (Donlan, 2002; Tuson and Weibel, 2013). The physicochemical properties of a substratum surface can affect bacterial attachment and how quickly biofilms develop, including surface roughness, hydrophobicity, surface charge, and presence of conditioning films (Donlan, 2002; Srey et al., 2013).

The relationship between bacterial attachment and surface roughness has been reported for years. However, opinion is divided regarding the effect of roughness on bacterial attachment and biofilm formation. Some studies revealed that the irregularities of abiotic surfaces promote bacterial attachment and biofilm development due to lower shear forces and larger surface area to which bacterial cells can attach on rougher surfaces (Pedersen, 1990; Bollen et al., 1997; Donlan, 2002; Yu et al., 2016), whereas a contradictory result showed that surface roughness had no influence on bacterial attachment (Vanhaecke et al., 1990; Flint et al., 2000; Zhao et al., 2014). The opposite results may be due to different extracellular structures and physicochemical properties of different bacteria as well as the diverse physicochemical properties of a substratum surface with varied hydrophobicity, surface charge and conditioning films.

Surface hydrophobicity, the strongest long range non-covalent interactions in biological systems, has been thought to play an important role in bacterial attachment. Hydrophobic surfaces seem to be easier for bacteria to colonize than hydrophilic materials (Teixeira and Oliveira, 1999; Donlan and Costerton, 2002; Sousa et al., 2011). This is probably because hydrophobicity reduces repulsive forces between the bacterial surface and colonization substratum. Yu et al. (2016) attributed hydrophobicity and surface roughness of substratum to the early attachment of *Streptococcus mutans.* Teixeira and Oliveira (1999) also reported the positive correlation between the degree of hydrophobicity of polymeric substrate materials and the number of attached *Alcaligenes denetrificans.* A notable exception, however, is that *L. monocytogenes* is likely to attach to hydrophilic substrates such as stainless steel than hydrophobic surfaces like polytetrafluoroethylene (PTFE; Chavant et al., 2002). This might be due to the fact that attachment of bacterial cells is also influenced by bacterial surface hydrophobicity, which in turn depends on bacterial growth rate, bacterial species, and growth medium (Vacheethasanee et al., 1998; Katsikogianni and Missirlis, 2004). Vacheethasanee et al. (1998) observed that *S. epidermidis* strains with higher surface hydrophobicity attached to a greater extent than the ones with less surface hydrophobicity to polyethylene (PE). Studies have shown that hydrophobicity affects attachment of spores to surfaces, and that the more hydrophobic a surface or bacterium, the stronger the attachment (Husmark and Rönner, 1992; Faille et al., 2002). Husmark and Rönner (1992) reported that the hydrophobicity of *B. cereus* spores and hair like appendages surrounding the spores influenced attachment to inert surfaces. Hydrophobicity of bacteria can be determined by bacterial adherence to hydrocarbons (BATH) also currently known as microbial adherence to hydrocarbons (MATH), hydrophobic interaction chromatography (HIC), and contact angle measurements (Ukuku and Fett, 2002; Palmer et al., 2007). The choice of bacteria to attach to hydrophobic or hydrophilic surfaces depends on the structures and complex physiological and biochemical characteristics of both bacteria and their contacting surfaces.

Surface charge is another physical factor that affects the adhesion of bacteria to substratum. It is widely believed that most bacterial cells have a net negative surface charge due to the presence of considerable amount of carboxyl, amino, and phosphate groups (Dziubakiewicz et al., 2013). Thus, surface that is positively charged promotes bacterial attachment while a negatively charged surface will encourage resistance to bacterial attachment (Tuson and Weibel, 2013). It should be further noted that the surface charge of bacteria differs between bacterial species and is influenced by growth medium, bacterial age, pH, and ionic strength (Katsikogianni and Missirlis, 2004). It is often describes by the zeta potential (Palmer et al., 2007). Studies investigating the influence of surface charge on the adhesion ability of *E. coli* to inert surfaces have shown positive relationship in some cases (Dickson and Koohmaraie, 1989; Ukuku and Fett, 2002) and no correlation in the other (Rivas et al., 2007). The discrepancies in these studies could be due to the employed methods, which utilized different growth media and buffers, to demine bacterial surface charge. Similarly, QS in *E. coli* causes an increase in the negative charge on cell surfaces, which in turn promote the association of bacteria with surfaces during the early phases of biofilm formation (Tuson and Weibel, 2013). Electrostatic interaction chromatography (ESIC) has been widely used to measure bacterial surface charge (Ukuku and Fett, 2002).

Nearly all bacteria moving from liquid media toward surfaces make their first contact with conditioning films. The films are essential in the bacterial adhesion process and are formed as a result of adsorption of nutrient molecules onto the material surfaces which lead to changes in physicochemical characteristics of the surfaces and in turn affect the bacterial attachment (Lorite et al., 2011). These films are formed within minutes of exposure with concomitant growth for several hours (Donlan, 2002).

### Irreversible Adhesion

The irreversible attachment is attained through the effects of short range interactions such as dipole-dipole interactions, hydrogen, ionic and covalent bonding, and hydrophobic interactions with involvement of bacterial structural adhesions (Bos et al., 1999). The surface of bacteria is gifted with different adhesins that are projected away from the cell surface into the extracellular environment (Berne et al., 2015). So far, adhesive structures of bacteria, including flagella, pili/fimbriae, and non-fimbrial adhesions, were identified to be involved in the development of biofilms (Berne et al., 2015). The presence of these surface organelles help bacterial cells to make first physical contact with substrates (Petrova et al., 2012; Berne et al., 2015; Carniello et al., 2018). Flagellum is a whip like filamentous appendage concerned with bacterial locomotion (Haiko and Westerlund-Wikstrom, 2013). Flagella driven motility can either be swimming (in liquids) or swarming (on solid moist surfaces). Various species of bacteria exhibit both type of movements to navigate bacterial cells toward a favorable environment and to attach onto a surface (Kearns, 2010; Hintsche et al., 2017). Numerous studies have reported the importance of flagella mediated motility in early attachment and subsequent biofilm formation. Flagella can initiate the adhesion of cells to surfaces by overcoming the repulsive forces that might hinder cell to surface interactions (Van Houdt and Michiels, 2005; Terashima et al., 2008; Lemon et al., 2007; Haiko and Westerlund-Wikstrom, 2013; Wood, 2013). Non-flagellated mutants of *L. monocytogenes* were impaired in surface adhesion compared to the wild type with short incubation periods. However, with longer times of incubation, surface coverage by non-flagellated mutant cells almost reach the same level as flagellated cells, suggesting that the presence of flagella is crucial for initial and early attachment (Vatanyoopaisarn et al., 2000).

Pili/fimbriae are also filamentous appendages used for bacterial attachment to each other and early cell-surface attachment (Konto-Ghiorghi et al., 2009; Maldarelli et al., 2016). For example, *P. aeruginosa* can employ a pilus mediated form of bacterial surface movement called twitching motility (Alarcon et al., 2009). In *K. pneumoniae, Streptococcus agalactiae, Clostridium difficile*, and *Acinetobacter baumannii*, pili play important roles in their early attachment to surfaces (Konto-Ghiorghi et al., 2009; Maldarelli et al., 2016; Pakharukova et al., 2018). Type 1 and type 3 fimbriae on the surface of *K. pneumoniae* facilitate attachment on abiotic surfaces and formation of mature biofilm, while only type 1 fimbriae initiate attachment of *E. amylovora* on abiotic surfaces and biofilm formation (Di Martino et al., 2003; Murphy et al., 2013). And the wild type of *E. amylovora* attached in greater numbers to surfaces than the mutant type with a deletion in type I fimbriae, which suggests the importance of adhesion structures in the formation of mature biofilms (Koczan et al., 2011). Additionally, thin aggregative fimbriae, also called curli fimbriae and antigen 43, are found to enhance initial surface attachment of bacteria (Heras et al., 2014; Carter et al., 2016). Moreover, distinct adhesins in some bacteria might be used to mediate transition from transient to permanent surface attachment. For example, formation of the monolayer in *Caulobacter crescentus* is mediated by a strong adhesive polysaccharide called the holdfast (Karatan and Watnick, 2009). Another example is polysaccharide intercellular adhesin (PIA) produced by *S. epidermidis* that is essential for cell to cell attachment and subsequent biofilm development (Rohde et al., 2010).

Bacterial pathogens also generate special adhesins that enable them not only adhere to receptors on eukaryotic cell surface but also facilitate their internalization. For instance, *Yersinia pseudotuberculosis* and *Yersinia enterocolitica* produce a protein invasin which adheres to β1 integrins on the surface of M-cells and causes crossing of *Yersinia* into M-cells (Bonazzi et al., 2009; Karatan and Watnick, 2009).

A cell-to-cell signaling mechanism called QS also coordinate individual cells to initiate formation of bacterial biofilms (Abraham, 2016). Using QS, bacteria synthesize and release first messengers like chemical signals (autoinducers, AIs) to enable cell-to-cell communication within bacterial population (Li and Tian, 2012; Papenfort and Bassler, 2016). Both Gram-negative and Gram-positive bacteria employ cell-to-cell signaling mechanisms to regulate biofilm formation. Gram-negative bacteria primarily used acyl homoserine lactones (AHLs), whereas Gram-positive bacteria used oligopeptides, universal AIs that can be utilized by both Gram-negative and Gram-positive bacteria (Miller and Bassler, 2001; Sperandio et al., 2001; Sun et al., 2004).

### EPS Production

Irreversible adhesion is progressed through the production of EPS regulated by QS of the resident bacterial cells. Bacteria synthesize and secrete EPSs which are an essential component of biofilm extracellular matrix. EPS can mediate both cohesion of bacteria and adhesion of biofilms to surfaces via hydrophobic interactions and ion bridging interactions (Fahs et al., 2014; Costa et al., 2018). Overall, EPS plays critical roles in adherence to surfaces, cell–cell recognition, biofilm formation, biofilm structure, retention of water, signaling, protection of cells, symbiosis with plants, trap of nutrients, and genetic exchange (Dogsa et al., 2005; Limoli et al., 2015; Flemming, 2016; Costa et al., 2018). In addition, secondary messenger c-di-GMP is regarded as one of the stimuli for the transition from reversible to irreversible adhesion through the production of EPS and cell surface structures (Toyofuku et al., 2016).

The main constituents of EPS, including polysaccharides, proteins, DNAs, lipids and other polymeric compounds, depend on the bacterial species, and the environmental conditions (Myszka and Czaczyk, 2009; Kostakioti et al., 2013; Limoli et al., 2015; Jayathilake et al., 2017; Bacosa et al., 2018; Costa et al., 2018). Polysaccharides are a major constituent of the EPS matrix and necessary for biofilm development and growth in most bacteria (Flemming et al., 2016). In Gram-negative bacteria, the polysaccharides are usually neutral or polyanionic. The anionic property is considered to be as a result of the presence of uronic acids or ketal-linked pyruvates. This is thought to facilitates association of divalent cations such as magnesium and calcium, which are very important for cross-linking of polymer strands leading to greater binding force in a developed biofilm formation (Donlan, 2002). In Gram-positive bacteria such as *Staphylococci*, however, the EPS is mainly cationic (Donlan, 2002). EPS matrix also contains considerable amounts of proteins such as enzymes and proteinaceous structures like pili and fimbriae. Besides, DNA is an integral part of EPS matrix which acts as an intercellular connector (Flemming et al., 2016). Lipids found in the matrix also play important roles for the attachment of *Thiobacillus ferrooxidans* (Flemming et al., 2016).

### Biofilm Maturation

At this phase, the genetic machineries of EPS such as a 15 gene-long *epsA-O* cluster concerning biofilm formation in *Bacillus subtilis* become activated when intensity of the AIs exceed certain threshold. Bacteria continue to multiply within embedded EPS matrix by using the AIs signals, and conduct to formation of microcolonies and maturation of biofilms (Lopez et al., 2010; Toyofuku et al., 2016). Following microcolony formation and EPS accumulation, changes in gene expressions are induced, and the products of these genes are utilized for the production of EPS that act as biological “glue” between embedded bacterial cells (Frederick et al., 2011; Karimi et al., 2015). The formation of matrix is followed by formation of water-filled channels which act like circulatory systems, conveying nutrients to the cells communities and removing unwanted products (Garnett and Matthews, 2012). Structural analysis of the microcolonies often shows a pyramid/mushroom-shaped multicellular structure (Garnett and Matthews, 2012). During the process of maturation, motility is restricted within the microcolonies as the production of bacterial surface structures is inhibited, and the gene expression pattern of the sessile cells differs significantly from the planktonic cells. For example, more than 57 biofilm associated proteins, that were not present in the planktonic cells, have been detected in *P. aeruginosa* microcolony (Hall-Stoodley and Stoodley, 2002). Moreover, QS enables communication among bacteria of the same or different species through secretion and detection of AIs. Bacteria use these signaling molecules to sense the presence of each other and to regulate gene expression in response to changes of their population density (Kaplan, 2010; Guttenplan and Kearns, 2013; Wei and Ma, 2013; Berlanga and Guerrero, 2016). Herein, AIs have an important role in maintaining existing biofilms.

### Dispersal/Detachment

The biofilm detachment process, also known as dispersal, represents the terminal process of biofilm development. It is regarded as a strategy of bacterial cells to leave biofilms and continue another biofilm life cycle (Singh et al., 2017). That is, dispersal of surface attached cells from biofilms is a naturally program phenomenon which allows bacterial cells to form new microcolonies on other fresh substrates in response to particular physiological or environmental conditions (Diaz-Salazar et al., 2017). Dispersal is a complex process regulated by environmental signals, signal transduction pathways, and effectors (Kaplan, 2010).

Although the dispersal mechanisms vary among bacteria, the whole process can still be divided into three common stages: detachment of cells from the microcolonies, movement of cell to a fresh substrate, and adhesion of the cells to the new substrate (Kaplan, 2010; Shen et al., 2018). Furthermore, the detachment can be an active action (i.e., seeding) that cells in biofilms initiate the detachment of themselves in response to changes in their environment such as antimicrobial stress, matrix-degrading enzymes and nutrient starvation, or passive behaviors (i.e., sloughing and erosion) mediated by external forces such as shear forces (Kaplan, 2010; Fleming and Rumbaugh, 2017; Lee and Yoon, 2017). In other words, seeding dispersal is the active detachment mechanism associated with rapid release of microcolonies or planktonic cells from the center of the biofilm, leaving an empty hollow cavity; Sloughing is the sudden detachment of a large portion of a biofilm; Erosion is a release of small portion bacteria from the biofilm. *Aggregatibacter actinomycetemcomitans, P. aeruginosa, Serratia marcescens*, and *S. aureus* can exhibit seeding dispersal of biofilms (Kaplan, 2010; Lee and Yoon, 2017).

During active dispersal, genes involved in cell motility, such as flagella synthesis and EPS degradation are usually up-regulated, while genes related to EPS production (i.e., polysaccharide synthesis), attachment, and fimbriae synthesis are often down-regulated (Kostakioti et al., 2013). Another effective way to disperse biofilm is to inhibit the c-di-GMP signaling pathways because reduction of intracellular c-di-GMP levels will either inhibit biofilm development or enhance biofilm dispersal (Kaplan, 2010). Furthermore, environmental factors like temperature change, pH, nutrients, and oxygen deficiency can contribute to biofilm dispersal (Kostakioti et al., 2013). For example, limited oxygen supply facilitates biofilm detachment by promoting c-di-GMP degradation. An increase in glucose supply can decrease intracellular c-di-GMP, resulting in the raise of flagella synthesis that eases detachment process (Lee and Yoon, 2017). Moreover, there are various physicochemical parameters and inherent cell properties such as autolysis that facilitate biofilm dispersal (Kaplan, 2010; Kostakioti et al., 2013; Lee and Yoon, 2017).

## Regulating Approaches for Bacterial Biofilms

Unlike the planktonic bacteria, biofilms are not effectively eliminated by ordinary cleaning, washing and disinfection methods (LeChevallier et al., 1988; Somers and Wong, 2004). The formation of biofilm, however, can also play beneficial roles (Morikawa, 2006; Singh et al., 2006; Edwards and Kjellerup, 2013; Naidoo and Olaniran, 2013; Singh et al., 2019). Therefore, multiple factors have also been explored to promote formation of beneficial biofilms (Ansari et al., 2012). Herein, there are different strategies developed to prevent, control or promote bacterial biofilm development, which are closely related to the regulation of bacterial attachment, signal transduction (quorum sensing interference), and bacterial biofilm matrix (Table 4; Chung and Toh, 2014).

**TABLE 4:** The regulating approaches for bacterial biofilms.

**Strategy**	**Mechanism**	**References**
**1. Prevention and control as well as promotion of bacterial attachment**		
1.1 Antifouling surfaces		
Poly ethylene glycol (PEG)	Bacteria repelling coatings	Roosjen et al., 2003; Roosjen et al., 2005
1.2 Antimicrobial surfaces		
Silver	Antimicrobial releasing coatings	Bazaka et al., 2012; Francolini et al., 2017
quaternary ammoniumcompounds (QACs)	Contact killing coatings	Hasan et al., 2013; Achinas et al., 2019
1.3 Small molecules		
aryl rhodanines	Anti-adhesion	Cegelski et al., 2009; Chung and Toh, 2014
Pilicides and curlicides	Anti-adhesion	Cegelski et al., 2009; Chorell et al., 2012
1.4 Surface modification		
Oxygen plasma on carbon based materials	Promotion of bacterial attachment, biofilm formation and electricity generation in BESs	Flexer et al., 2013
Nitrogen plasma on carbon anode	Promotion of biofilm formation and electricity production in MFCs	He et al., 2012
Polyethylene membrane (PE) modified with positively charged graft polymer chains (diethylamino)	High adhesiveness for nitrifying bacteria than original unmodified membrane and rapiddevelopment of nitrifying biofilms	Hibiya et al., 2000
Methoxy-PEG-amine (-PEG-NH_2_) modification on a rough PP surface and the smooth PE surface	Enhancement in biofilm formation	Lackner et al., 2009
**2. Control or promotion of bacterial signal transduction (quorum sensing interference)**		
2.1 Quorum quenchers (QQs)		
Enzymes includinglactonase, acylase, oxidoreductase, and paraoxonase	Enzymatic degradation of signal molecules	Sadekuzzaman et al., 2015
2.2 Quorum sensing inhibitors (QSIs)		
N-octanoyl-L-HSL (C8-HSL)	Inhibition of the synthesis of signal molecules	Hirakawa and Tomita, 2013
2.3 Natural agents		
Furanone, ajoene, naringin, musaceae, andcurcumin	Prevention of bacterial biofilm	Ponnusamy et al., 2010; Musthafa et al., 2010; Jakobsen et al., 2012; Truchado et al., 2012; Packiavathy et al., 2014
Honey	Restriction to biofilm development	Sharahi et al., 2019
2.4 AIs and QS genes		
10 μM acyl homoserine lactones	Encouragement of beneficial biofilm formation	Chen et al., 2017
100 μM quinolone	Enhancement in biofilm mass	Chen et al., 2017
increased expression of QS genes *lasI* and *rhlI*	Improvement of biofilm formation and EPS production	Mangwani et al., 2016
**3. Disruption of bacterial biofilm matrix**		
3.1 Matrix targeting enzymes		
DNase I, restriction endonucleases, glycoside hydrolases, proteases, and dispersin B	EPS degradation	Kaplan, 2014; Parrino et al., 2019
3.2 Bacteriophages		
phage SAP-26	EPS degradation	Lu and Collins, 2007
3.3 Small molecules		
Cis-2 decenoic acid (C2DA)	Biofilm dispersal	Jennings et al., 2012; Chung and Toh, 2014

### Prevention and Control as Well as Promotion of Bacterial Attachment

Inhibition of cell attachment is an ideal approach to prevent biofilm formation at an initial phase. Therefore, remodeling the surface or coating the surface with the substances that do not encourage the bacterial adhesion could probably impede establishment of bacterial biofilm (Rogers et al., 1994; Chung and Toh, 2014).

Antibiofilm surfaces can mainly be divided into antifouling surfaces and antibacterial ones. The former prevents bacterial attachment onto the surfaces while the latter kills bacteria on the surfaces (Xu et al., 2005; Li et al., 2018). Coating agents and paints such as silver, titanium oxide, grapheme, arsenic, mercury oxide, copper oxide, and zinc oxide nanoparticles have been developed and used effectively as antifoulants (Kuang et al., 2018). Recently, poly(ethylene glycol) (PEG) has been the most widely used antifouling coatings in the marine and biomedical industries (Zhang et al., 2017). Surfaces covered with PEG have been shown to resist the adhesion of bacteria, because of the hydrophilic surface property. PEG coatings are able to repel quite a number of bacterial species like *S. aureus, S. epidermidis, P. aeruginosa*, and *E. coli* (Roosjen et al., 2003; Roosjen et al., 2005). Antibacterial surfaces are designed for indwelling medical devices (e.g., catheters and endotracheal tube), which can be colonized by biofilm forming bacteria, to release antibiotics, bacteriocins, metal ions, plant extracts or nanoparticles against pathogens such as *S. aureus*, *Candida albicans, P. aeruginosa*, and *E. faecalis* (Dror et al., 2009; Chung and Toh, 2014; Sanchez et al., 2016; Kuang et al., 2018; Vasilev et al., 2018). Inhibition of biofilm formation can be achieved on medical device surfaces through coating with silver (Bazaka et al., 2012; Francolini et al., 2017). The mechanism of action of silver-based materials is mainly related with the release of silver ion (Ag^+^) from the surface, and the required amount for an optimal effect ranging from 10 μM to 10 μM (Bazaka et al., 2012; Francolini et al., 2017). Similarly, quaternary ammonium compounds (QACs) are widely used as antibacterial agents for contact killing coatings. Contrary to the antibiotic-release mechanism of silver ions, QACs coatings have a long-lasting contact based antimicrobial mechanism (Hasan et al., 2013; Achinas et al., 2019). Unfortunately, contact killing surfaces have the drawback that some microorganisms are able to develop resistance against these surfaces (Hasan et al., 2013). What’s more, small molecules like aryl rhodanines can prevent the early phases of biofilms formed by Gram-positive pathogens by inhibiting bacterial adhesion to surfaces (Cegelski et al., 2009; Chung and Toh, 2014). And small synthetic compounds pillicides and curlicides can interfere with bacterial adhesion by inhibiting production of bacterial pili/fimbriae and curli (Cegelski et al., 2009). Natural products honey and tea can also inhibit bacterial attachment (Kuang et al., 2018; Sharahi et al., 2019).

On the other hand, attachment of beneficial bacteria can be promoted by surface modification of materials, which includes both physical and chemical based modification, or electrochemical oxidation treatment (Berlowska et al., 2013; Flexer et al., 2013; Kang et al., 2014).

The surface characteristics can be designed and altered to enhance bacterial attachment and formation of beneficial biofilms for BESs and yeast fermentation industry (Upadhyayula and Gadhamshetty, 2010; Berlowska et al., 2013). The use of nitrogen or oxygen plasma on carbon based materials such as graphite electrodes has been shown to increase surface energy and hydrophilicity which in turn promotes bacterial attachment, biofilm formation and electricity generation in BESs (Flexer et al., 2013). Also, the use of nitrogen plasma on carbon anode can alter surface roughness and hydrophobicity to promote biofilm formation and electricity production in MFCs (He et al., 2012). In addition, carbon felt electrodes treated with UV/O3 can enhance *Shewanella oneidensis* MR-1 attachment and biofilm formation, leading to increased electron transfer rate and greater current density production in MFCs (Cornejo et al., 2015).

The conversion of ammonium to nitrate (nitrification) is an essential process in wastewater treatment (Lackner et al., 2009). However, the organisms responsible for nitrification have very low growth rates and do not form strong biofilms (Lackner et al., 2009). Hence, efforts need to be made to maintain these nitrifiers in reactor systems (Busscher et al., 1995). It has been proposed that the bond between the attaching bacteria and the materials surface is determinant on biofilm strength and shear resistance (Busscher et al., 1995). Various approaches have been used to promote attachment of nitrifying bacteria onto a membrane surface (Hibiya et al., 2000; Terada et al., 2004; Lackner et al., 2009). Hibiya et al. (2000) observed that the PE membrane whose surface is modified with positively charged graft polymer chains (diethylamino) exhibited a high adhesiveness for nitrifying bacteria than original unmodified membrane, and nitrifying biofilms develop rapidly. Lackner et al. (2009) modified PE and polypropylene (PP) membranes to improve the attachment and shear resistance of nitrifying biofilms. They used a combination of plasma polymerization and wet chemistry to introduce chains of PEG containing two different functional groups (-PEG-NH_2_ and -PEG-CH_3_) to the membrane surfaces. They demonstrated that the methoxy-PEG-amine (-PEG-NH_2_) modification on a rough PP surface and the smooth PE surface had a clear enhancement in biofilm formation. The amino group of methoxy-PEG-amine acts as an attractive force for nitrifiers like *Nitrosomonas europea* and *Nitrobacter winogradskyi*, which enhanced formation of biofilm.

### Control or Promotion of Bacterial Signal Transduction (Quorum Sensing Interference)

Bacterial QS depends upon a series of events such as production of signal, signal dissemination, signal receptors, signal detection, gene expression, and signaling response. Therefore, quorum quenchers (QQs), or quorum sensing inhibitors (QSIs) that interfere with these processes might potentially inhibit bacterial QS and ultimately biofilm formation (Li and Tian, 2012; Remy et al., 2018). The quorum-quenching approach employing quorum quenching enzymes to inactivate quorum sensing signals is important in healthcare settings and medicine as well as industrial membrane bioreactors, crop production and aquaculture (Fong et al., 2018). Quorum quenching enzymes, lactonase, acylase, oxidoreductase, and paraoxonase, have been discovered in various species of bacteria (Chen et al., 2013). The well-known mechanism of action of QQs is the inactivation of acyl homoserine lactone molecules (Sadekuzzaman et al., 2015). Another mechanism is the inhibition of the synthesis of signal molecules (e.g., AHLs) by QSIs such as N-octanoyl-L-HSL (C8-HSL) that prevents the enzymatic activity of Lux operon proteins (Hirakawa and Tomita, 2013). The natural QSIs known to prevent bacterial biofilm mainly include furanone (Ponnusamy et al., 2010), ajoene (Jakobsen et al., 2012), naringin (Truchado et al., 2012), musaceae (Musthafa et al., 2010), and curcumin (Packiavathy et al., 2014). Moreover, a natural ingredient honey at elevated amount can interfere with genes involved in bacterial communications such as AI-2 and LsrA, thereby limiting biofilm development (Sharahi et al., 2019). Furthermore, the presence of a secondary messenger c-di-GMP in elevated amount promotes biofilm formation in bacteria. Therefore, inhibiting the c-di-GMP pathway may be effective method to prevent biofilm formation (Sharahi et al., 2019).

On the other hand, genetic engineering of AIs can encourage beneficial biofilm formation, which plays critical roles in power generation, wastewater treatment and bioremediation (Mangwani et al., 2016; Chen et al., 2017). For example, a decrease in start-up time from 10 days to 4 days in dual chamber MFC was achieved by adding a kind of AIs (10 μM AHLs; Chen et al., 2017). An AI (100 μM quinolone) enhances biofilm mass of extremophile *Halanaerobium praevalence* on the anode of MFC, leading to effective treatment of high salinity wastewater and improved power generation (Chen et al., 2017). Furthermore, QS bacteria can degrade a broad range of pollutants (Mangwani et al., 2015). The marine *P. aeruginosa* N6P6 biofilm formation and EPS production can be improved by increasing the expression of QS genes *lasI* and *rhlI*, which contributes to increase rate of polycyclic aromatic hydrocarbon degradation (Mangwani et al., 2016).

### Disruption of Bacterial Biofilm Matrix

To disperse bacterial biofilms, it’s essential to destroy the structural components of EPS (Flemming and Wingender, 2010; Wei and Ma, 2013). Thus, degradation of the EPS matrix can be effective method to interfere with bacterial biofilm formation.

EPS matrix-degrading enzymes, including deoxyribonuclease I (DNase I), restriction endonucleases, glycoside hydrolases, proteases, and dispersin B, can inhibit bacterial biofilm formation and facilitate dispersion of established biofilm colonies (Kaplan, 2014). As soon as the biofilm matrix is enzymatically degraded, the bacterial cells are then released as planktonic cells which are easily eliminated by various antibacterial agents, disinfectants, phages, or immune systems (Kaplan, 2014; Parrino et al., 2019).

Phages can cross the EPS matrix by either diffusion or with the assist of phage-derived enzymes (Sao-Jose, 2018; Simmons et al., 2018). A genetically engineered lytic phage having a biofilm degrading enzyme showed more efficient eradication of biofilm than non-enzymatic phage (Lu and Collins, 2007). Combined with antibiotic rifampicin, the phage SAP-26 was able to cross the biofilm matrix leading to disruption of biofilm architectures (Hughes et al., 1998; Rahman et al., 2011). Cis-2 decenoic acid (C2DA) is a medium-chain fatty acid chemical messenger produced by *Paeruginosa* to initiate the dispersion of established bacterial biofilms C2DA not only effectively induced biofilm dispersal but may also inhibits initiation of biofilm formation (Jennings et al., 2012; Chung and Toh, 2014).

Therefore, EPS matrix can be destructed with enzymes and phages. As the research progresses, more EPS degradation methods will be found.

## Conclusion

Bacterial biofilm formation occurs in sequential and well-regulated events and is the predominant bacterial lifestyle in most natural and man-made environments. The ability of bacteria to colonize surfaces and to establish biofilms are considered serious issues and has been associated with detrimental consequences in many branches related to food, water, pharmacy and healthcare. In an effort to get rid of harmful biofilms, various techniques and approaches have been developed which were mostly concerned with interference against bacterial attachment and QS as well as biofilm matrix destruction. However, bacterial biofilms affect the environments beyond risk. There are numerous beneficial applications of bacterial biofilms. Biofilm-associated bacteria play essential roles in the transformation of hazardous pollutants to harmless substances, the protection of plants against phytopathogens, the plant growth promotion, as well as the removal of excess nutrients from wastewater. Moreover, beneficial biofilms formation can be encouraged in many industrial and environmental areas through surface modification and QS signals.

## Future Perspectives

Researches on the harmful effects of biofilms on healthcare, agriculture, food industry, drinking water, and oceans will continue to dominate the research field in the near future. However, with the deepening of people’s understanding of the dual-sidedness of the role of biofilms more than risks, the researches focusing on plant protection, bioremediation, wastewater treatment, and corrosion control of biofilms will be increasing. In addition, with the application of next-generation technologies such as various omics to biofilm researches, new bacterial biofilm regulation mechanisms are expected to be discovered. Therefore, researches of biofilms may be carried out in the following aspects in the future:

(1) Control of bacterial biofilms that are harmful to human society;
(2) Utilization of beneficial bacteria with high-production biofilms;
(3) In-depth investigation of regulatory mechanisms for the formation and dispersion of bacterial biofilms, especially researches related to beneficial biofilms;
(4) Elucidation of interaction mechanisms of bacterial biofilms with inanimate or living body interfaces;
(5) Development of new commercial products based on bacterial biofilms;
(6) Exploration of application schemes for bacterial biofilm products.

It is believed that through the unremitting efforts of researchers, biofilms will play more and more important roles in both basic researches and practical applications in recent years.

## Author Contributions

The idea of this manuscript was conceived by TH. MM wrote the manuscript. AI, XF, YG, YY, and XJ helped to analyze the literatures. TH, XG, and JQ reviewed the manuscript.

## Conflict of Interest

The authors declare that the research was conducted in the absence of any commercial or financial relationships that could be construed as a potential conflict of interest.
